# Nutritive Value and Physical Properties of Neo-Tropical Rodent Meat-with Emphasis on the Capybara (*Hydrochoerus hydrochaeris*)

**DOI:** 10.3390/ani10112134

**Published:** 2020-11-17

**Authors:** Anwar Jamaal Ali, Kegan Romelle Jones

**Affiliations:** 1 Department of Food Production (DFP), Faculty of Food and Agriculture (FFA), St. Augustine Campus, University of the West Indies (UWI), St. Augustine, Trinidad and Tobago; anwaarjali@gmail.com; 2 Department of Basic Veterinary Sciences (DBVS), Faculty of Medical Sciences (FMS), Mt. Hope Campus, School of Veterinary Medicine (SVM), University of the West Indies (UWI), Mt. Hope, Trinidad and Tobago

**Keywords:** *Hydrochoerus hydrochaeris*, *Dasyprocta leporina*, *Agouti paca*, agouti, lappe, capybara

## Abstract

**Simple Summary:**

This review highlights the nutritive value of meat from neo-tropical rodents that have the potential to be domesticated. In a resource depleting world these species can serve as a potential source of animal protein to decrease poverty and hunger. Major emphasis was placed on the capybara as its meat was the one most investigated amongst the other rodents. The meat of these rodents is very nutritious, having high concentrations of protein and polyunsaturated fatty acid (PUFA). This meat is also low in cholesterol, fats, and saturated fatty acids (SFA). In human diets high levels of cholesterol, fat, and saturated fatty acids have been related to chronic lifestyle diseases. Capybaras that were raised in an environment with access to a pond had higher levels of PUFAs and lower levels of SFAs. There is little information on the other neo-tropical rodents including the lappe (*Agouti paca*) and the agouti (*Dasyprocta leporina*). Thus, the new horizon in research will include the meat analysis of agouti and lappe meat. Further investigations can be done on the effects that diet and the environment can have on the composition of neo-tropical rodent meat.

**Abstract:**

This review will focus on the nutritive characteristics of meat from neo-tropical rodents which have not yet been domesticated. These rodents include the capybara (*Hydrochoerus hydrochaeris*), agouti (*Dasyprocta leporina*), and the lappe (*Agouti paca*). Information about the meat characteristics of these rodents were obtained from peer reviewed journal articles. Literature was obtained using search engines such as Google Scholar, Uwi linc, and Pub Med Central. Keywords used in the searches were “capybara”, “*Hydrochoerus hydrochaeris*”, “agouti”, “*Dasyprocta leporina*”, “*Agouti paca*/*Cuniculus paca*”, lappe”, and “meat proximate analysis”. Over four decades of literature was searched, spanning from the 1970s to 2020. There is a vast amount of information on the meats of the capybara, but limited information on the lappe, and there is a dearth of information on the agouti. Capybara meat is considered to be highly nutritious, with high levels of polyunsaturated fatty acids (PUFA), low levels of saturated fatty acids (SFA), low levels of fats and cholesterol. The animals that were reared in conditions which were similar to their habitat had lower levels of SFA. Diet, age, confinement, and sex had an effect on the chemical composition of the meat of the capybara. On average, capybara meat had a moisture content of 75%, protein 22%, ash 1.5%, and lipid 1%. During the authors’ search of the literature, no information was found on the proximate composition of the lappe or agouti meat. Information regarding the fatty acid profile of lappe meat was found, as well as a description of the physical characteristics of agouti and lappe meat. The physical parameter shows that the lappe has the most tender meat of the three rodent species, while the agouti has the least tender meat, and the capybara being intermediary. Neo-tropical rodent meat is highly nutritious, and is an excellent protein alternative for the growing population of the world. These animals are adapted to challenging environments and can grow well using locally available feed resources. However, further research needs to be conducted on the proximate analysis on lappe and agouti meat to fully inform consumers about its nutrient value.

## 1. Introduction

Non-domestic neo-tropical animals have tremendous potential as a source of meat for human consumption. Rodents such as the lappe (*Agouti paca*), agouti (*Dasyprocta leporina*), and the capybara (*Hydrochoerus hydrochaeris*) have been identified as having great potential to be domesticated [1]. These rodents have been used in rural villages by hunters as a source of meat protein [2]. These animals are ideal for sustainable agriculture as they have the ability to utilize locally available feedstuff and convert them into animal protein. They can be fed locally available forages and feed by-products for maintenance [3]. The agouti (*D.leporina*) is considered by some authors as an opportunistic feeder [4], others have considered these animals as omnivores as they can consume both plant and animal matter [5,6]. The lappe, agouti, and capybara possess a large cecum which gives them the ability to digest fibrous feeds. The lappe is considered a frugivore with the majority of its diet comprising of fruits and seed [7]. However, the capybara is described as a herbivore and is better able to utilize fibrous feeds than cattle [8]. These rodents are hindgut fermenters which practice cecotrophy.

The agouti has tremendous reproductive potential, having a gestation period between 104 to 120 days [9]. The agouti has the potential to produce three litters per year and one to three offspring per parturition [1,10,11,12]. Thus, one mated pair can produce up to six offspring per year. The adult agouti can weigh 2–5 kg and females obtain sexual maturity at nine months with males becoming sexually mature by twelve months [10,13,14]. Investigators reported that the agouti had a dressing percentage of 57% [13]. These animals can be reared in captivity to produce a sustainable source of meat for subsistence farmers. The agouti is considered a pest to crop farmers and animals are obtained from the wild for intensive production [10,15].

The lappe (*A. paca*) has a mature body weight between 6–14 kg [10,16,17]. It usually produces one offspring per parturitions with two parturitions per year [10,16]. The gestation period of the lappe ranges between 138–172 days [10], 152–156 days [18]. Litter intervals have been reported to range from 247–266 days [16] and have a similar timeframe for sexual maturity as the agouti (9 months for females and 12 months for males) [10]. At present, there are no records in the literature of the dressing percentage and meat yield in the lappe. This is an area that is in desperate need of investigation.

The capybara (*H. hydrochaeris*) is the largest rodent on earth. Mature body weight ranges from 60 to 100 kg [19]. These animals have a dressing percentage of 62.47% [19]. The average litter size ranges from 3.3 [20] to 3.8 [21] but animals can produce as much as eight offspring per litter. Two litters can be attained per year if the inter birthing period is 180 days [21]. In previous studies, the inter birthing period was found to be 251 days [20]. Sexual maturity is attained 26 months of age [20]. The capybara has the potential to produce 16 offspring per female per year. Of the three neo-tropical rodents that are being investigated, the capybara has the greatest potential reproductive efficiency, followed by the agouti, and then lastly, the lappe.

With an increasing world population, the demand for meat is also increasing [9]. Conventional sources of meat such as pigs, poultry, and cattle have been shown to have a devastating effect on the environment [22]. Livestock farming is the number one contributor of methane and has the greatest use of freshwater as an industry [22]. Unconventional meat sources, such as bush meat, have been shown to be a much safer alternative and are much more efficient in its resource usage and waste management [23].

The intensive production of the agouti can help with its conservation as well as provide a means of its sustainable production [1]. It can also be economically viable and is more conservative than the extensive production of livestock [1]. It reduces the need for hunting which decreases the stress of the wild animals from hunting pressure [1]. Furthermore, this increases the natural fauna of the forested areas (habitats of the animals), which helps to balance the ecosystem, which contributes to the idea of stability and conservation of the environment [24]. The agoutis are reared intensively in breeding colonies, they utilize local feed resources (fruits, seeds, and forages) [1]. The production system that is adopted is integrated with crops and soils. The crop residues are fed to the agouti and the feces of the agouti are used as fertilizer for the soil [1]. If these animals are reared intensively for a long period of time and controlled breeding is done, these animals may become domesticated in the not too distant future. Therefore, these species that are considered as bushmeat can be a healthier option for human consumers.

Bush meat, especially those of rodents, has been shown to be a very healthy source of food [23]. While considered distasteful by most in Western societies, it is widely accepted in many cultures around the world and has been a source of food for centuries [23,25]. However, in recent years, a paradigm shift has occurred, and bush meat is becoming more accepted and occupies a place in niche markets as exotic meats. This requires the producers as well as consumers to have knowledge on the quality factors of their products, in this case, bush meat specifically of the capybara, agouti, and lappe.

Thus, the objective of this paper was to discuss the literature on meat composition from neo-tropical rodents with the potential to be domesticated and the agouti, lappe, and capybara fall within this category.

## 2. The Case for Bush Meat

The world population is increasing and is expected to reach 10 billion by the year 2050 [22]. As countries become increasingly developed, there is an increase in the protein intake per capita [22]. Conventional sources of meat are largely derived from intensive farming practices which cause a huge strain on the environment [22]. Coupled with this, land availability for livestock production is becoming increasingly more limited. More efficient usage of this land as well as more effective management of the animal species involved are both required.

The utilization of wild species is being considered as an alternative method as these species thrive in the local conditions and contribute to the natural environment which they inhabit. This bush meat is a source of protein for the rural and low-income earning communities. It also plays an important role in the agro tourism industry as seen in South Africa where bush meat consumption is seen as an authentic “African experience” by tourists [23].

Expert opinions have suggested the necessity for the contribution of rodent meat as a means of sustainability but the acceptability of the meat may not be forthcoming as these animals may be viewed as rodent [25]. However, this review does not focus on the acceptability of this meat source but summarizes the nutritive value. It is even suggested that these species which are hunted as game meat be farmed intensively in the near future [25]. Farming of these wild species also removes the constrains of overhunting [23]. Rodents have a great potential to be a viable source of protein as a farmed wildlife species. While the nutritional value of these species is not well known, the commercial farming of them opens up the possibility of this research [23]. There is a severe lack of research done on rodent meat even in areas of the world where they are consumed regularly [25].

In South America, bush meat acts as a cheap food resource and a source of protein for rural areas. While in the past it acted as a main source of protein, this is no longer true as conventional meats are cheaper and more readily available [26]. However, it has been shown that consumption of bush meat persists as it provides a distinct taste which serves as a delicacy [26]. Some species even provide by-products which are useful to the communities. In recent years, commercial farm rearing of these animals has become a new prospect as there is a demand in local markets as well as foreign exotic meat markets [27].

The capybara (*Hydrochoerus hydrochaeris*), agouti (*Dasyprocta leporina*), agouti paca (*Cuniculus paca*), and guinea pig (*Cavia porcellus*) have been suggested to have immense production potential in Latin America [28,29]. Guinea pigs have been an important protein source for Native Indians in South America for centuries. Native Indians have developed their own husbandry practices and are well capable of farming these animals as a livestock source [28]. For centuries, they have served its meat as a ritualistic practice and nowadays, the locals in the Andes partake in its consumption. It is considered a high value meat and is inculcated in their holiday tradition. It also has value as a means of bush medicinal remedies [30].

The capybara is considered healthier than other red meats due to its high crude protein values, low fat, and cholesterol. High humidity and temperature can be limiting for conventional livestock species, but this animal can thrive in these conditions. It has been shown to be very capable of surviving in intensive production and has a low maintenance cost as it can feed on fibrous material such as cassava roots and peel [31,32]. While rabbit (*Oryctolagus cuniculus*) meat is not considered bush meat, it is still an unconventional source of meat and is not as popular as “red meats” and poultry. Studies show that the potential for rabbit meat as consumable towards younger generations is high [33]. In order to increase the appeal, information and services must be done targeting potential consumers along the lines of price, convenience of products, and nutritional value [33]. The same can be applied to rodent meat as it is very similar to rabbit meat because rabbit meat is considered a healthier meat being low in cholesterol and high in protein.

## 3. Proximate Analysis of Neo-Tropical Rodent Meat

A summary of the proximate analysis of capybara meat is given in Table 1. The meat composition of the capybara (*H. hydrochaeris*), lappe (*A. paca*), and agouti (*D. leporina*) will be discussed in this section. Adult capybaras reared in individual cages fed napier grass and pelleted ration (containing corn and soybean meal) had meat cuts with 75.8% moisture, 21.74% protein, 0.74% lipids, 0.9% ash, and 23 mg/100 g cholesterol of intramuscular fat [31]. Capybara meat has similar protein content as both rabbit and guinea pig meat but differences were seen in lipid content. In general, rabbit meat has protein content ranging from 18.7–22.1% and lipid content ranging from 1.2–12.8% [33,34]. Rabbit meat contained more cholesterol (48–60 mg/100 g) when compared to capybara meat [34]. Guinea pig meat had similar protein content to capybara (22.7–24.8%) but had higher fat content (2.97–4.51 g/100 g) [35]. Guinea pigs also had similar dressing percentage to the capybara and (ranging from 55–67.4%) [36,37,38,39]. Girardi et al. [31] investigated the chemical composition of the capybara meats reared in different conditions. The animals were slaughtered at nine months (20 kg), these animals were fed forages and supplemented with pelletized diet for rabbits. There were no differences recorded for the moisture, protein, and ash for different meat (loin and ham) and rearing conditions (enclosures with and without ponds). However, there were differences seen in lipid content on commercial cuts (loin 1.81–2.26% and ham 3.93–4.74%). Capybaras that were reared with a pond in its enclosure had lower cholesterol values (45.7–45.9 mg/kg) compared to animals without a pond in their enclosure (51.9–52.1 mg/kg) [31]. The commercial meat cuts of the capybara showed differences in moisture and cholesterol. The shoulder had the highest moisture content, the loin and chop had the lowest moisture content, whilst the ham and chest had intermediate moisture content. Cholesterol values were lowest in shoulder as compared to the other meat cuts [40]. In general, capybara meat can be considered healthier than rabbit and guinea pig meat as it has similar protein content but lower fat and cholesterol content. In recent times, healthy meat is considered as those which contain high levels of protein, low levels of fat, low levels of saturated fatty acid, and high levels of polyunsaturated fatty acids.

Bressan et al. [40] quantified the lipid content of various meat cuts of captive reared adult capybara as 0.85% with no significant difference in the lipid content of commercial cuts. In contrast, further work done found that the loin had the lowest fat content, the ham, ribs *L. dorsi*, and bottom sirloin having the highest fat content. The brisket of the capybara had intermediate values for fat [19]. The mineral content of the capybara meat was also investigated, with significant differences noted in the meat cuts for iron content. On average capybara meat had 0.03% calcium, 0.2% phosphorus, and 0.0005% iron. The ribs had the highest iron content (0.0007%) and the *L. dorsi* having the lowest iron content (0.0004%) [19]. Jardim et al. [41] noticed the effect of sex and slaughtering method on chemical parameters in capybara meat. The *L. dorsi* muscle was analyzed and there were no differences observed on the effect of sex on moisture, protein, ash, and cholesterol. In contrast, female capybaras had higher level of lipids in comparison to males [41,42,43] and meat from female carcasses had higher levels of cholesterol [43].

**Table 1:** Proximate analysis of the meat products and edible offal of capybara (*H. hydrochaeris*).

Physiological State	Diet and Housing	Moisture (%)	Protein (%)	Lipid (%)	Ash (%)	Cholesterol (mg/100 g)	Source
Adult males and females	Captive reared in individual cages fed forages and rabbit pellets	75.8	21.74	0.74	0.9	23	[31]
Adult males and females	Captive reared animals	-	-	0.85	-	-	[40]
Adult female	Captive reared fed forages only	-	-	1.09	-	-	[42]
Adult male				0.65			
Adult males and females	Free ranging animals with diet consisting of cultivated crops	74.75	20.65	1.48	1.2	-	[19]
Adult male and females	-	76.59	20.04	0.91	0.9	-	[44]
Adult male and female (smoked meat products)	-	67.72	24.93	2.77	2.16	-	
Adult male and female (*L. dorsi*)	Free ranging	75.7	22.8	0.4	1.1	-	[45]
Adult male and female (*Semimembranosus*)	Free ranging	76.5	22.1	0.3	1.1	-	
Adult male and female (liver)	Free ranging	74.1	22	0.4	1.3	-	
Adult male and female (heart)	Free ranging	77.4	20.1	0.2	0.9	-	
Adult male and female	Captive reared fed forages with rabbit pellets	75.87	22.11	0.98 (males) 1.75 (females)	1.09	28.1	[41]
Adult males	Free ranging	-	-	1.5	-	35	[43]
Adult females	Free ranging	-	-	1.75	-	47.5	

Roca et al. [44] investigated the proximate composition of raw meat as well as smoked meat products of capybara. The capybara meats were cured and smoked, the curing mixture consisted of consisted of water, common salt, refined sugar, polyphosphates, sodium nitrite, natural flavoring, and sodium erythorborate. The pieces were subjected to dry heat of 50 °C for one hour and smoking for six hours, with cooking carried out in direct steam. The smoked meat products showed different chemical composition in comparison to the raw meat. The smoked meat had a reduction in moisture (67.72%) and increases in protein (24.93%), lipids (2.77%), and ash (2.16%) [44]. There were no significant differences recorded in the taste, strange taste, and strange aroma of the smoked meats. However, the smoked meats had higher values in tenderness and juiciness [44].

The *L. dorsi*, *Semimembranosus*, liver, and heart of the capybara were analyzed. *L. dorsi* had similar chemical properties as the *Semimembranosus* muscle. However, visceral organs such as the liver had chemical properties that were dissimilar to the muscle with the heart having the highest moisture content and lowest protein content [45]. In the literature search that was conducted, there were no published articles on the chemical composition of agouti (*D. leporina*) or lappe (*A. paca*) meat. However, the moisture content for the leg cut in the agouti was 78.99% (Ali and Jones unpublished). This is an area in desperate need of attention due to the enormous potential these animals have as a protein source for rural communities. Some work was published on the physical properties of the meats of lappe and agouti as well as the fatty acid profile of lappe meat. These properties will be discussed in a subsequent section of this document.

## 4. Fatty Acid Content of Neo-Tropical Rodent Meat

Several authors have investigated the fatty acid composition of capybara and lappe meat. In their investigations, enquiries were made on that diet, housing, and commercial cuts had on fatty acid concentrations. Betancourt and Jair Diaz [46] analyzed meat of lappe and capybara for fatty acid composition. The capybara was found to have palmitic acid in highest concentration; this was followed by oleic, eicosatetraenoic acid, and linoleic acid. The total amount of saturated fatty acid ranged from 39.4–39.54% and polyunsaturated fatty acids were 29.04–32.7%. Thus, the polyunsaturated to saturated fatty acid ratio ranged from 0.73–0.82. The total amount of omega-3 fatty acids were between 20–23.4% and omega-6 was 5.63–12%. The ratio of omega-6 to omega-3 ratios was between 0.24–0.6 [41,46]. Therefore, guinea pigs that were not fed flaxseed supplements had lower nutritive value than capybara meat. Rabbit carcass had similar saturated (38.6%) and polyunsaturated (23%) composition as muscle from capybara meat but the hind legs of the rabbit had higher saturated and polyunsaturated fatty acids [34]. Jardim et al. [41] observed the effects of sex and slaughter on the semimembranosus muscle. There were no differences noted in the fatty acid composition of the meat tissue of either sexes or slaughter technique. The major concentrations of fatty acids were palmitic acid, oleic acid, linoleic acid, and stearic acid [41].

Commercial cuts of capybara meat were also investigated to ascertain the various fatty acids present. Differences in fatty acids were found between cuts for palmitic acid, palmitoleic acid, oleic acid, arachidonic acid, and adrenic acid [40]. Palmitic acids (C16:0) was the fatty acid with the highest concentration (ham, chest, loin, shoulder, and chop). Linoleic acid (C18:2 n-6) was second in the fatty acid concentration, followed by oleic acid (C18:1 n-9), and arachidonic acid (C20:4 n-6) [40].

The concentration of saturated fatty acids for commercial cuts was as follows: ham (38.36%), chest (46.32%), loin (51.90%), shoulder (45.90%), and chops (41.37%). The polyunsaturated fatty acids were: ham (43.32%), chest (24.89%), loin (67.51%), shoulder (32.86%), and chops (32.97%). Therefore, the polyunsaturated to saturated fatty acid ratios were: ham (1.13), chest (0.54), loin (1.30), shoulder (0.72), and chops (0.8) [40]. The omega-3 contents in the ham were 1.6%, chest 1.69%, loin 2.00%, shoulder 1.44%, and chops 1.57%. The omega-6 contents in the ham were 37.11%, chest 18.48%, loin 28.99%, shoulder 26.92%, and chops 27.12%. Therefore, the omega-6 to omega-3 fatty acid ratios were 0.04 (ham), 0.09 (chest), 0.07 (loin), 0.05 (shoulder), and 0.05 (chops) [40]. The data above shows that capybara meat cuts have higher levels of polyunsaturated fatty acids in comparison to saturated fatty acids. The data also shows that there is a higher content of omega-3 in comparison to omega-6 fatty acids. This shows that the meat of capybara is healthy for human consumption due to its low level of saturated and high levels of omega-3 fatty acids.

The highest concentration of fatty acids found in the meat was linoleic acid, palmitic acid, stearic and oleic acid [42]. Investigators also found no difference in fatty acid profile between sexes or ages. Further work done on the effect the environment has on fatty acid profiles in the capybara revealed that animals without ponds had higher levels of saturated fatty acids (myristic acid, palmitic acid, and stearic acid). The capybara reared with ponds had higher polyunsaturated fatty acid content in their meat (oleic acid and linoleic acid) [31]. The total saturated fatty acids in animals without a pond were 40.00% whilst those with a pond in their enclosure were 32.6%. Therefore, animals without ponds had a lower polyunsaturated to saturated fatty acid ratio [31] (Table 2).

The meat of the lappe (*A. paca*), which is the second largest rodent in the world, was evaluated for fatty acid composition. The fatty acids in greatest concentrations were palmitic acid (C16:0), oleic acid (C18:1 n-9), linoleic acid (C18:2 n-6), and alpha linolenic acid (C18:3 n-3) [46]. Total saturated fatty acids for lappe meat were 36.3%, whilst polyunsaturated fatty acids were 22.5%. Thus, the ratio of polyunsaturated fatty acids to saturated fatty acid for lappe meat was 0.62. The total omega-3 fatty acids were 22.5% in contrast to the omega-6 fatty acids which was 13.3%. Thus, the omega-6 to omega-3 ratio for the lappe was 0.5 [46].

Factors such as the environment (without a pond) can decrease the level of polyunsaturated fatty acid and increase the level of saturated fatty acids. Therefore, when capybaras are to be reared in captivity for human consumption, these animals should be given an environment with a pond. This will enrich the animals’ enclosure as well as produce meat that is healthier for human consumption. The large differences that can be seen in the total omega-3 to omega-6 fatty acids can be attributed to the different environments in which the animals were grown as well as the different cuts that were sampled in the various studies (Table 2). In the literature that was search, there was no record of the fatty acid profile for agouti (*D. leporina*) meat. However, the lappe and capybara meat had similar profiles of fatty acid composition.

## 5. Physical Parameters of Neo-Tropical Rodent Meat

Table 3 summarizes the physical properties capybara, agouti, and lappe meats. Limited information has been recorded in the literature on the physical parameters of neo-tropical rodent meat. However, authors have noted some physical parameters of agouti (*D. leporina*) meat. The L* value (brightness or hue) for the leg and shoulder was found to be 49.28 and 42.34, respectively. The a* value (red color) for the leg and shoulder was found to be 5.28 and 10.78, respectively. The b* value (blue to yellow color) for the leg and shoulder was found to be −0.47 and 2.1, respectively (Ali and Jones unpublished). The author noted that the shoulder had higher parameters (brightness, red color, blue to yellow color, and firmness) than the leg cuts of meat for the agouti and this is an intermediate value in comparison to domesticated livestock.

The firmness value for the leg and shoulder was found to be 10.87 and 14.36 kg, respectively. The shear force for the leg cut and shoulder was found to be 86.58 Newtons (N) and 109.4 N, respectively. The capybara was found to have physical meat parameters that were different to the agouti. The capybara had a lower value for brightness (L*) (39.94), a higher value for red color (a*) (22.10), and a higher value for the blue to yellow pigment (b*) (11.12) [19].

With respect to the shear force of the meat, all three rodents varied greatly. However, comparisons made between these animals based on tenderness cannot be made as variability may have occurred in the age, slaughter techniques, and environment in which each species of animal was raised. Authors reported 2.59 N for the ham, 2.56 N for the shoulder, and 2.51 N for the loin [47]. These values were similar to those reported for the commercial cuts of capybara meat and the *L. dorsi* and *semimembranosus* (2.84 N). In contrast, authors found that the *L. dorsi* and the *semimembranosus* in capybara was tougher (11.6 N and 9.16 N) when the Warner Bratzler shear force analysis was used [45]. Sensory work was done using human evaluation on the lappe meat. Using human taste panel, there were no differences noticed in the commercial cuts with respect to the color, odor, flavor, and softness [45]. As the author used qualitative methods of analysis, further work can be done on lappe meat using quantitative methods of analysis.

## 6. Conclusions

Neotropical rodents with the potential for domestication such as the lappe, agouti, and capybara have enormous potential to supplying healthy meat protein for human consumption in the neo-tropical. There is a dearth of information on the chemical and physical characteristics of the lappe and agouti meat. Due to its increasing popularity, there is reasonable information on the meat qualities of the capybara. Lappe and capybara meat seems to be very nutritious for human diets, having a high concentration of polyunsaturated fatty acids in the meat and having high concentrations of omega-3 and omega-6 fatty acids. The composition of capybara meat seemed to be affected by sex, with females having higher concentration of cholesterol and lipid in their meat and by environment, with animals having enclosure with ponds having higher concentrations of polyunsaturated fatty acids and lower concentrations of saturated fatty acids compared to their counterparts without ponds. The capybara meat has high concentrations of protein (average 22%) but this information was lacking for the agouti and the lappe. The physical parameters appeared to be different for each rodent species, with the lappe having the softest meat and the agouti having the toughest meat.

## Figures and Tables

**Table 2:** Fatty acid composition of capybara and lappe meat.

Animal	C16:0 (%)	C18:2 n-6 (%)	C 18:1 n-9 (%)	C20:4 n-6 (%)	C20:3 n-3 (%)	C18:0 (%)	Total SFA (%)	Total PUFA (%)	PUFA: SFA	Total Omega-3 (%)	Total Omega-6 (%)	Omega-6 to Omega-3 Ratio	Ref.
*H. hydrochaeris*	27.8	10.7	24.5	-	20.0	-	39.4	32.7	0.82	20	12	0.6	[46]
*H. hydrochaeris* *(semimembranosus)*	29.57	19.19	27.87	-	-	6.57	39.54	29.04	0.73	23.41	5.63	0.24	[41]
*H. hydrochaeris* (Ham)	27.64	23.21	17.97	12.81	-	-	38.36	43.32	1.13	1.60	37.11	0.04	[40]
*H. hydrochaeris* (Chest)	35.23	12.10	35.74	5.79	-	-	46.32	24.89	0.54	1.69	18.48	0.09	
*H. hydrochaeris* (Loin)	42.02	16.77	29.31	11.18	-	-	51.90	67.51	1.30	2.00	28.99	0.07	
*H. hydrochaeris* (Shoulder)	34.21	20.28	26.27	5.87	-	-	45.90	32.86	0.72	1.44	26.92	0.05	
*H. hydrochaeris* (Chop)	32.07	17.52	26.95	8.80	-	-	41.37	32.97	0.80	1.57	27.12	0.05	
*H. hydrochaeris* (Loin)	16.38	18.78	10.91	-	-	11.13	-	-	-	-	-	-	[42]
*H. hydrochaeris (reared without pond)*	24.75	24.85	26.45	-	-	6.54	40.00	28.1	0.7	-	-	-	[31]
*H. hydrochaeris (reared with pond)*	22.2	27.35	29.1	-	-	4.73	32.6	31.7	0.97	-	-	-	
*Agouti paca*	26.6	13.3	21.6	-	-	8.4	36.3	36.1	0.99	22.5	13.3	0.59	[46]

**Table 3:** Physical parameters for selected neo-tropical rodent meats.

Animal	Color (L*)	Color (a*)	Color (b*)	Firmness (kg)	Shear Force (N)	Ref.
*D.leporina* (leg)	49.28	5.28	−0.47	10.87	86.578	Ali and Jones unpublished
*D. leporina* (shoulder)	42.34	10.78	2.1	14.36	109.411	
*H. hydrochaeris* (commercial cuts)	39.94	22.10	11.12	-	2.84	[19]
*H. hydrochaeris* (*L. dorsi*)	-	-	-	-	11.6	[45]
*H. hydrochaeris* (*Semimembranosus*)	-	-	-	-	9.14	
*A. paca (ham)*	-	-	-	-	2.59	[47]
*A. paca (shoulder)*	-	-	-	-	2.56	
*A. paca (loin)*	-	-	-	-	2.51	
